# Cancer Stem Cells Therapeutic Target Database: The First Comprehensive Database for Therapeutic Targets of Cancer Stem Cells

**DOI:** 10.5966/sctm.2015-0289

**Published:** 2016-10-11

**Authors:** Xiaoqing Hu, Ye Cong, Huizhe (Howard) Luo, Sijin Wu, Liyuan (Eric) Zhao, Quentin Liu, Yongliang Yang

**Affiliations:** ^1^ Center for Molecular Medicine, School of Life Science and Biotechnology, Dalian University of Technology, Dalian, People's Republic of China; ^2^ Institute of Cancer Stem Cell, Dalian Medical University, Dalian, People's Republic of China; ^3^ Sun Yat‐sen University Cancer Center, State Key Laboratory of Oncology in South China, Collaborative Innovation Center of Cancer Medicine, Guangzhou, People's Republic of China

## Abstract

Cancer stem cells (CSCs) are a subpopulation of tumor cells that have strong self‐renewal capabilities and may contribute to the failure of conventional cancer therapies. Hence, therapeutics homing in on CSCs represent a novel and promising approach that may eradicate malignant tumors. However, the lack of information on validated targets of CSCs has greatly hindered the development of CSC‐directed therapeutics. Herein, we describe the Cancer Stem Cells Therapeutic Target Database (CSCTT), the first online database to provide a rich bioinformatics resource for the display, search, and analysis of structure, function, and related annotation for therapeutic targets of cancer stem cells. CSCTT contains 135 proteins that are potential targets of CSCs, with validated experimental evidence manually curated from existing literatures. Proteins are carefully annotated with a detailed description of protein families, biological process, related diseases, and experimental evidences. In addition, CSCTT has compiled 213 documented therapeutic methods for cancer stem cells, including 118 small molecules and 20 biotherapy methods. The CSCTT may serve as a useful platform for the development of CSC‐directed therapeutics against various malignant tumors. The CSCTT database is freely available to the public at http://www.csctt.org/. Stem Cells Translational Medicine *2017;6:331–334*

**Significance Statement**

Although the definition and role of cancer stem cells (CSCs, also called tumor‐initiating cells) remain a topic of much debate, increasing evidence suggests that CSCs may be the driving force behind chemotherapy/radiotherapy resistance, as well as metastasis. Consequently, the elimination or differentiation of CSCs is critical for treating malignant tumors and improving clinical outcomes. Unfortunately, the progress of research into the development of anti‐CSC therapeutics has been rather slow, and no anti‐CSC drugs are yet in clinical use. Hence, there is an urgent need to develop a database that compiles useful information for putative targets and therapeutic methods associated with CSCs.

## Clinical Relevance of Cancer Stem Cells for Eradicating Malignant Tumors

The war on conventional cancer therapy has mostly failed because of the intrinsic resistance to drugs/radiation, as well as uncontrolled cancer progression 1, 2. In the past decades, increasing evidence suggests that the failure of existing therapies to eradicate malignant tumors may be attributed to cancer stem cells (CSCs), often defined as a small population of tumor cells that are capable of self‐renewal and differentiation 3, 4. Importantly, elevated CSC levels in some tumor types are closely associated with higher tumor grade and poor clinical outcome 5. Moreover, it has been demonstrated that traditional anticancer drugs and radiation therapy 6 are ineffective against CSCs. This is because traditional therapeutic methods only shrink or reduce the bulk of cancerous cells for temporary relief but do not touch the CSCs. Therefore, CSCs represent viable and clinically relevant targets for eradicating malignant tumors 7.

To date, numerous CSC‐directed approaches hold promise for a long‐term cure for malignant tumors. In particular, direct targeting of CSC‐expressed molecular markers or CSC‐related pathways by small molecule compounds (monoclonal antibody [mAb] and small hairpin RNA) has been widely exploited to ablate CSCs in malignant tumors. For instance, ABCB5 has been identified as a molecular marker of melanoma CSCs, and administration of anti‐ABCB5 mAb can substantially inhibit tumor formation and neoplastic progression 8. Moreover, the mAb‐targeting epithelial cell adhesion molecule expressed by breast and colon CSCs has been translated into clinical therapies 9, 10.

On the other hand, indirect strategies, such as homing in at targets of the microenvironment related to CSCs, have also proved quite useful. For example, Jin et al. reported a mAb therapy targeting the adhesion molecule CD44, which regulates cell‐cell contact in a microenvironment 11. Remarkably, administration of this mAb to NOD/SCID mice transplanted with human acute myeloid leukemia cells can significantly reduce neoplastic repopulation. Other indirect strategies that are useful for eradicating CSCs include therapeutic methods targeting angiogenesis 12, differentiation, and epigenetic mechanisms 13, 14.

Collectively, given the strong capabilities of CSCs to initiate tumorigenesis and promote metastasis, therapeutic strategies targeting CSCs have become a very promising and appealing approach to treat and eventually cure cancer 15. Hence, a bioinformatics resource that compiles validated targets and therapeutic methods for CSCs will help researchers discover molecules or biologics that selectively kill CSCs or make CSCs vulnerable to chemotherapy or radiotherapy.

## Design and Web Interface of Cancer Stem Cells Therapeutic Target Database

The Cancer Stem Cells Therapeutic Target Database (CSCTT) consists of two major types of data: (a) therapeutic targets for CSCs and (b) therapeutic methods for CSCs. Information on potential targets for CSCs was collected from the scientific literature and various web resources, such as the Protein Data Bank 16, PubChem 17, Kyoto Encyclopedia of Genes and Genomes (KEGG) 18, and UniProt 19. The criteria used to select the publications for building CSCTT were as follows: We used the combination of multiple keywords, such as “cancer stem cell AND drug OR molecule” and “tumor initiating cells AND drug OR molecule” to search PubMed. Subsequently, 1,069 reports with abstracts were obtained from PubMed and the full text was downloaded for manual curation. The criteria used to select the targets deposited in CSCTT were as follows: After manual curation, approximately 122 proteins and microRNAs (miRNAs) as druggable targets of CSCs that had at least one report showing clear experimental evidence implicating the role in cancer stem cell, stem‐like cell lines, tumor‐initiating cell lines, and cancer progenitor cell lines were deposited into the CSCTT.

Herein, “druggable target” was defined as a biomolecule (protein or miRNA) that is implicated in CSCs and is known to bind with high affinity to a drug (small molecules, small interfering RNA, monoclonal antibodies; 50% inhibitory concentration <10 μM) with prior experimental evidence. Moreover, the binding of the drug to a CSC's druggable target must be able to eliminate or differentiate CSCs, with potential therapeutic benefit to patients by eradicating malignant tumors. This threshold was applied throughout the manual curation process. Each protein target was collected and carefully annotated with biological function, family and domain information, clear experimental evidence for its role in CSCs, related diseases, related pathways, and existing therapeutic methods if available. Additionally, ko numbers, which refer to the Genome Japan database, were added for some entries to highlight the significance of related KEGG pathways.

Meanwhile, we collected existing therapeutic methods targeting CSCs from the original literature, resulting in 213 available therapeutic methods that included 118 small molecule compounds, 75 drug combinations, and 20 biotherapy methods. In addition, the information for the related CSCs targets of the therapeutic methods was also curated from the references. We also annotated each molecule with chemical name, molecular structure, PubChem compound identifier, Chemical Abstracts Service registry number, and brief introduction of their inhibitory activities against CSCs. In recent years, cellular therapy has become an increasingly important approach to treat cancer. Noteworthy, we compiled 20 biotherapy methods from the available literature, such as chimeric antigen receptor T‐cell therapy, antibody‐based immunotherapy, oncolytic virus, and peptide‐based vaccines, with detailed mechanisms and references. We hope these will provide useful information that researchers can use to discover cellular therapeutics against CSCs.

**Figure 1:**
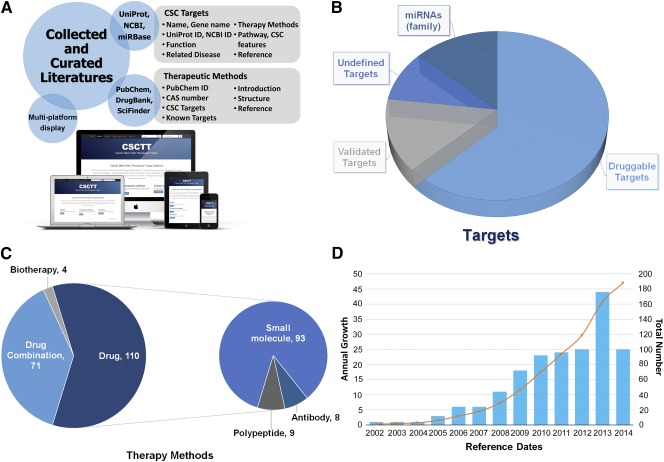
Workflow and statistics for CSCs targets and therapeutic methods deposited in CSCTT. **(A):** The fundamental framework of CSCTT. All results in CSCTT are deposited into an MySQL database and displayed in various browsers. **(B):** Category distribution of curated CSC targets. **(C):** Category distribution of available therapeutic methods for CSCs. **(D):** History of published reports on CSC targets. Abbreviations: CAS, Chemical Abstracts Service; CSC, cancer stem cell; CSCTT, Cancer Stem Cells Therapeutic Target Database; ID, identification number; miRNA, microRNA; NCBI, National Center for Biotechnology Information; UNP, UniProt.

CSCTT was designed as a relational database on a MySQL server (Fig. 1). The dynamic hypertext markup language (HTML) pages were compiled by hypertext preprocessor and generated in the web host. The web display of CSCTT was installed with the responsive web design method to adapt to different screen sizes in various web browsers. In addition, web access is enabled via HTML, cascading style sheets, and JavaScript framework. Firefox, version 3.6 or above (Mozilla, Mountain View, CA, https://www.mozilla.org), Safari (Apple, Cupertino, CA, http://www.apple.com), Chrome (Google, Mountain View, CA, http://www.google.com), and Internet Explorer, version 10 (Microsoft Corp., Redmond, WA, http://www.microsoft.com), were thoroughly tested and thus recommended for browsing CSCTT.

## Availability and Future Directions for CSCTT

As a useful resource with which to study the potential targets of CSCs and develop therapeutics against CSCs, the CSCTT database will be updated every 6 months, and the authors will respond to e‐mail request within 2 days. While the database update and developments continue, readers are welcome to e‐mail their comments, suggestions, or corrections, for which we will be very grateful. CSCTT can be accessed at http://csctt.org/.

## Conclusion

The rise of the CSC hypothesis indicates that the most effective treatment strategies to eradicate malignant tumors will be those specifically targeting CSCs 20. This is because CSCs are responsible for a few critical characteristics of malignant tumors, including metastasis and therapeutic resistance. Moreover, treatment strategies specifically targeting CSCs may have few side effects by avoiding the systemic or local toxicity that commonly occurs with traditional therapies. Finding such treatments targeting CSCs will require that we fully understand the existing targets as well as available therapeutic methods against CSCs. To this end, we developed CSCTT, a manually curated database dedicated to available targets and therapies for CSCs. It is the first online resource of this kind, and the data in CSCTT are freely available to all scientists working in the field of CSCs.

CSCTT provides users with a brief summary of protein targets and available therapeutic methods used for CSCs with documented evidence. The main advantage of using CSCTT over using a public search engine is that all the targets and therapies deposited in CSCTT are carefully compiled and include biological details from existing literature by manual curation. Moreover, CSCTT provides users with biological tools and the hyperlinks to various biological databases, including UniProt, Protein Data Bank, KEGG, and PubChem. CSCTT is convenient to use and will contribute to the understanding of CSCs in the formation and progression of malignant tumors. More importantly, the motivation for building CSCTT was to provide a useful resource for the development of therapeutics to eliminate or differentiate CSCs, and our laboratory has been investing research efforts in this direction. In recent years, it has been demonstrated that the combination of CSC‐targeted therapies with established chemotherapies or radiotherapies might have a synergistic action and thereby improve efficacy in clinical practice for cancer patients. We look forward to the development of more and more treatment methods targeting CSCs to improve the survival rate of cancer patients and eventually win the war against malignant tumors.

## Author Contributions

X.H.: collection and/or assembly of data, data analysis and interpretation, final approval of the manuscript; Y.C.: collection and/or assembly of data, data analysis and interpretation; H.H.L.: website design and construction; S.W.: conception and design, data analysis and interpretation, collection and/or assembly of data, final approval of the manuscript; L.E.Z.: collection and/or assembly of data; Q.L.: conception and design; Y.Y.: conception and design, manuscript writing, financial support, final approval of the manuscript.

## Disclosure of Potential Conflicts of Interest

The authors indicated no potential conflicts of interest.
